# Influence of vetiver root on strength of expansive soil-experimental study

**DOI:** 10.1371/journal.pone.0244818

**Published:** 2020-12-31

**Authors:** Gui-yao Wang, Yong-gang Huang, Run-fa Li, Jing-mei Chang, Jin-liang Fu

**Affiliations:** 1 School of Civil Engineering, Changsha University of Science and Technology, Changsha, P.R China; 2 China Railway Siyuan Engineering Croup Co., Ltd., Wuhan, P.R China; 3 CCCC First Highway Fifth Engineering Co., Ltd, Langfang, P.R China; 4 China CEC Engineering Corporation, Changsha, P.R China; University of Vigo, SPAIN

## Abstract

Grassroots have received more attention than the traditional method as soil reinforcement materials, especially the use of vetiver and other vegetation protection methods to treat expansive soil slope, have been tried and applied. To study the influence of grassroots on the strength properties of expansive soil, the laws of vetiver root growth over time and its vertical distribution of root content(*δ*) were firstly investigated by the experiment of planting vetiver. Then different *δ* and depth of planted soil were obtained. Simultaneously different *δ* and water content(*ω*) of grafted soil were made. With the direct shear test, the shear strength parameters of root-soil with different *δ* were analyzed. The shear test on root-soil composites with different *δ* was carried out to compare the strength characteristics of planted and grafted soil. The results showed that the *δ* of vetiver decreased with the increase of depth, and the *δ* of each layer increased with the growth period. The *δ* of 180d was 70.5% higher than that of 90d. The cohesion(*c*) of root-soil can be increased by more than 97%, and internal friction angle(*φ*) can be increased by more than 15.4% after 180 days. The *c* of 90 d vetiver root system can be increased by more than 18%, and the φ can be increased by more than 1.5%. At each depth, the *c* and φ of composite soil increases with the increase of *δ*, and the increment of cohesion (Δ*c*) and the increment of internal friction angle (Δ*φ*) increase with the increment of *δ*. But the increase in the ω will weaken the shear strength parameters of root-soil. Under the condition of the planted root system and grafted root system, the influence degree of *δ* on strength parameter of root-soil is different, and the law of strength parameters versus *δ* of grafted soil of 365d is similar to that of planted soil of 90d. And the root reinforcement of grafted soil is weaker than planted soil. Hence the grafted soil can´t accurately reflect the root-soil interaction of the existing root system.

## 1. Introduction

Expansive soil is a high-plastic clay soil that is rich in hydrophilic minerals and has evident expansion after water absorption and shrinkage after the loss of water. It is multi-fractured, also called “cracked soil”, and cracks have a significant impact on strength and slope instability [1]. It is necessary to analyze the causes of slope instability and propose new protection techniques. Therefore, the instability mechanism and treatment of expansive soil slopes have been widely studied [2–6]. Slope protection and reinforcement measures can be divided into three categories: surface water protection, slope surface protection, and support protection, most of which are used in combination of three [7]. As an important part of slope protection, ecological slope protection has been paid more and more attention.

Plants can increase the strength of soil [8–11] and improve the anti-disintegration performance of soil [8]. Therefore, grassroots could be applied to the treatment of expansive soil diseases. However, there are few reports on the treatment of expansive soil diseases with vegetation. Because of the particularity of expansive soil, for example, expansive soil tends to crack when it loses water and expands when it absorbs water. The treatment of expansive soil slope is different from the ordinary slope. Once the expansive soil slope is damaged, its influence is significant; hence its management method needs careful consideration. Recently, studies on the mechanism of soil consolidation of grass roots mainly concentrate on clay or silty soil [9–18], which not conducive to the innovation and advancement of ecological protection technology based on grass vegetation. It is also the significance of this study about effects of root on expansive soil.

Vegetation can be used to strengthen the slope mainly because the root system enhances the root-soil strength. About the effects of root on the strength of soil, some studies were carried out. The results showed that root could significantly enhance the shear strength of soils [9–11]. The diameter and distribution of roots had a significant effect on the *c* of the root-soil composite, but the influence on the φ was not evident [12]. Gonzalez-Ollauri and Mickovski found that soil roots increase the angle of internal friction by 20% compared to fallow soil [13]. The triaxial test of the root system, which was undertaken under undrained conditions, showed that the shear strength increased with the diameter of the root [14]. The larger the occupied area of the root in the sliding surface of root-soil, the greater the reinforcing effect of the root system on the soil [15]. The *c* of the root-soil gradually decreased from the surface to the deep of the earth [16]. Studies by Dan et al. [17] indicated the effect of reinforcement of root increases with the incmore significant ease of compactness. Zhang et al. [18] found that the correlation between shear strength and *δ* of root-soil complex was greater than its correlation with root length density or root-soil area ratio. However, people mostly focus on root parameters and ignore other factors besides the root system, such as water on root soil composite soil.

Due to vetiver has a strong vitality and a developed root system [19], it is commonly used to deal with slope stability problem of shallow [20]. In situ shear test on blocks of soil permeated with vetiver roots were carried out and showed a more excellent shear strength resistance than the samples of non-vegetated soil [21]. However, little literature has paid attention to its reinforced function on expansive soil. Guo [22], Bao et al. [23] discussed the characteristics of vetiver and black locust plants and the technical methods of planting, and compared the effects of other ways against slope landslide of shallow and its social and economic benefits and feasibility. The results showed that planting vetiver and hedgehog plants could enhance the slope stability and against landslides. Zhou et al. [24] carried out a confined expansion test and a direct shear test for expansive soils with different initial ω, results shown that the vetiver root system could reduce the expansive force and increase the shear strength. Expansive soil is easy to absorb water and swell; thus it generates swelling force and cracks. The increase of shear strength is helpful to restrain the cracking of expansive soil [25]. Hence the existence of the root system can alleviate the harm caused by this situation and is beneficial to the slope´s stability. And the key to the slope engineering of vetiver is to study whether the root system can enhance the shear strength of expansive soil. But soil sample in the experiment [24] was a grafted soil without considering the effect of actual planted soil.

In summary, there are many research works on the reinforced strength characteristics of root-soil. However, the study of soil-root reinforcement is methodologically challenging, and some aspects are difficult to validate. In addition, whether the remolded soil samples prepared by adding roots can accurately reflect the root-soil interaction of the existing root system, and the difference in the root-soil interaction between planted expansive soil and grafted soil, were rarely reported. In this paper, the effects of root systems at varying growth periods on shear strength of different soil layers during vetiver growth were studied. Furthermore, the relationship between shear strength parameters and *δ* of expansive soil with a grafted root system was studied. Our study has an important theoretical significance and engineering application value for applying vegetation to dispose of expansive soil slopes.

## 2. Materials and methods

### 2.1. Tested soil

#### 2.1.1. Ethics statement

Authors state that no specific permissions were required for these locations/activities. After we talked to the site construction unit, they agreed to give us some expansive soil. To ensure safety, the excavation process is completed by the builder and photographs are taken. In addition, the soil samples are sent to the laboratory by the construction unit's own pickup truck. We provide labor remuneration, hence there is no need for construction permit. In addition, vetiver is grown in a field near the lab and does not require a permit. Authors confirm that the field studies did not involve endangered or protected species.

**Fig 1:**
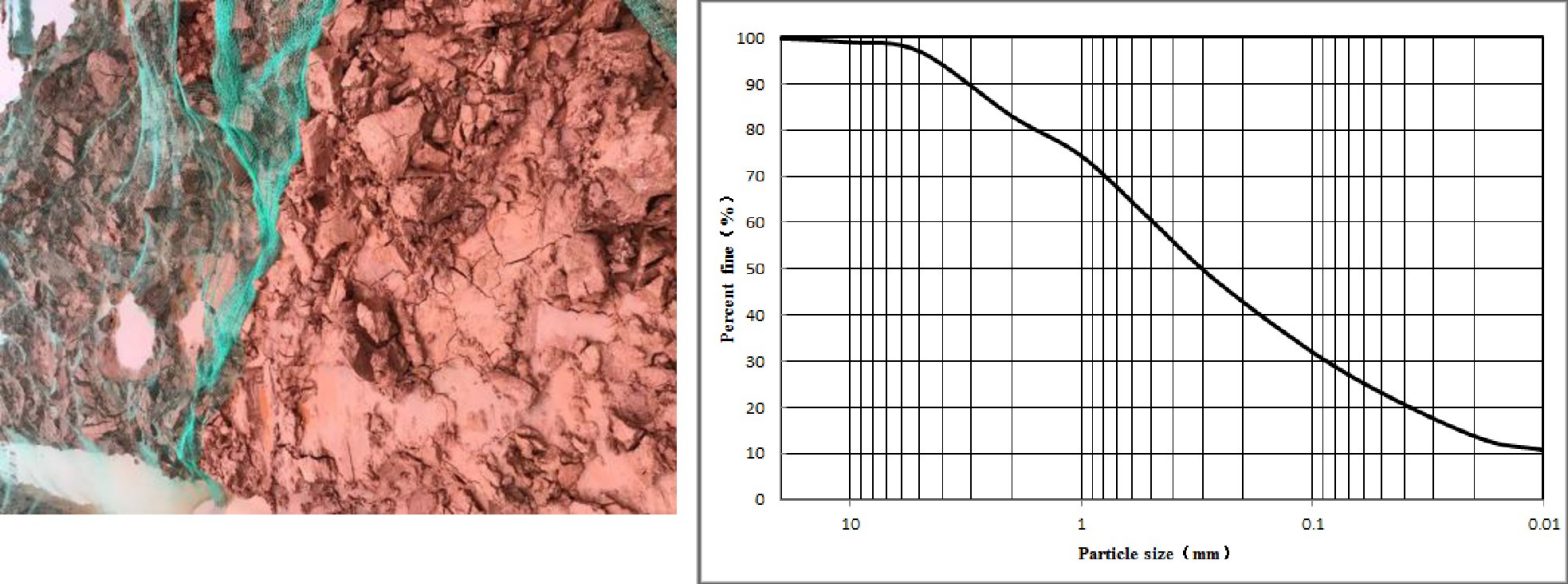
Expansive soil sampling site and gradation curves for the aggregates tested in this study.

The test soil was taken from an excavation slope (Fig 1) of Shuxiang Road (112.979566,28.121945), Changsha, China. Fig 1 also shows the aggregate gradation curves and fundamental properties of the soil used for the experiments. When the soil sample was taken back, it was air-dried and crushed and passed through a 2 mm sieve. After air-drying the *ω* was measured according to the Trade Standard of P.R. China (SL237-003 1999) to be 4%. The free expansion ratio, the maximum dry density, the optimum moisture content, the liquid limit moisture content, and the plastic limit moisture content were measured according to the Trade Standard of P.R. China (SL237-011 1999, SL237-007 1999, SL237-024 1999), and the basic physical parameters as shown in Table 1 were obtained. According to the test results and the specification for the design of highway subgrade (JTGD 030–2004), the soil sample was found to be weak expansive soil.

**Table 1:** Basic parameters of test soil sample.

Index	Optimum water content/%	Maximum dry density/ (g·cm^-3^)	Free swelling ratio/%	Plastic limit/%	Liquid limit/%	Plasticity index	Standard absorption water content/%
Value	20.5	1.56	50	23.9	53.5	29.6	3.2

### 2.2. Test preparation

Since the physical and mechanical properties of weak expansive soil are mainly affected by the density and ω, the test adopts three kinds of compaction degrees of 95%, 92%, and 90%. Each degree of compaction was controlled with a height of 5cm. The compacted mold was a high-strength transparent glass tube with an inner diameter of 19.8 cm and a height of 20 cm. After compaction, the lamination solidity from the bottom of the mold was 95%, 92%, and 90%, respectively. Taking 95% compaction as an example, the soil layer´s height was 5cm, and the *ω* was 20.5%. First, the required soil amount was calculated by formula (1)

$$m_{\mathrm{soil}}=\rho_{d\max}\lambda (1+\omega )$$ (1)

Where *m*_soil_ *is* the required soil amount, *ρ*_*d*max_ is the maximum dry density, *λ* is the compaction degrees, *ω* is the water content. *ρ*_*d*max_ can be obtained by compaction test, which is 1.56 g•cm^-3^.

**Fig 2:**
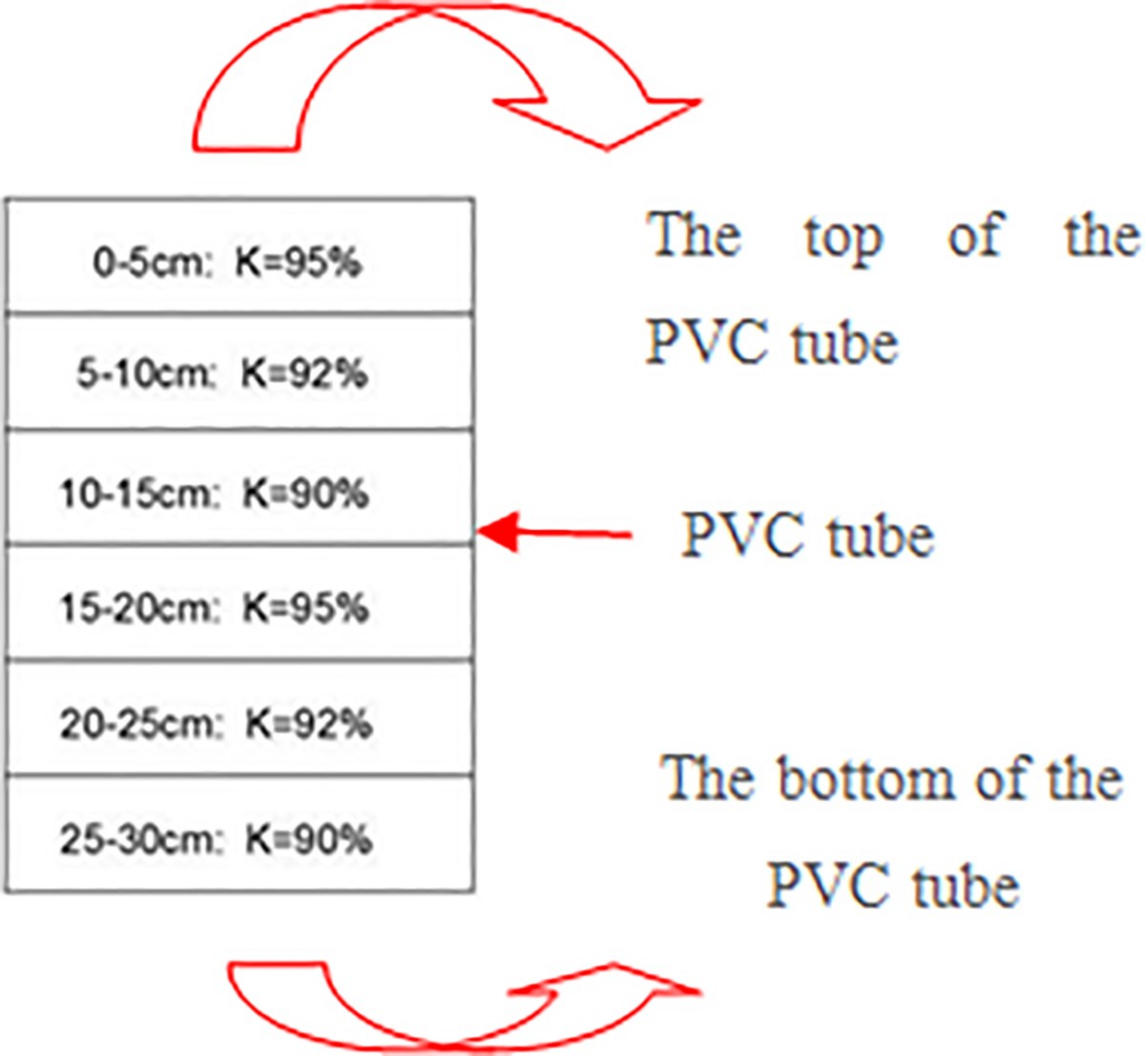
Compaction diagram.

**Fig 3:**
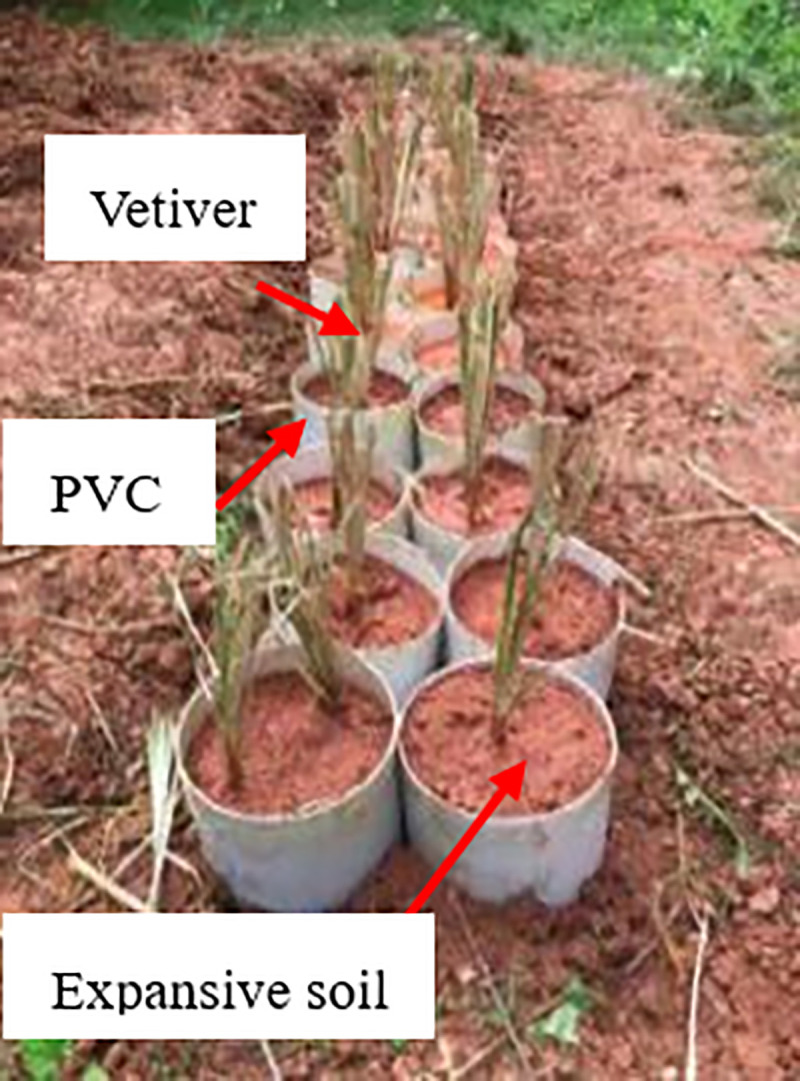
Vetiver cultivation.

Then the soil was added into the compacting mold twice to compact into a height of 5 cm. Referring to this method for the production of soil layers with different compactness, a 15 cm soil column was obtained according to this method. After the compaction, the 15cm high soil column was taken out from the mold, according to the 95% compaction soil layer on the top, and the 90% compaction soil layer was under the bottom, which was poured into the PVC pipe and then shaved on the upper surface. According to the same upper and lower order, a soil column was placed in the PVC pipe, and the soil column in the pipe was spliced into a solid of 30cm high (Fig 2); hence the high-pressure solid soil layer was close to the ground. To facilitate the removal of the root-soil composite, a crack was left in the PVC pipe, and the crack and the inner wall of the PVC pipe were taped. In addition, the bottom was sealed with a geotextile for draining. Since the compacted soil cannot directly transplant vetiver, 10cm high loose planting soil was covered the surface of the compacted soil. To ensure unity, 5~6 grass seeds were sowed in each PVC pipe. When the vegetation grew to 90, 180d respectively, planted soil was removed, and the compacted soil was retained. The root system of vetiver grass was developed into the compacted soil to form a root-soil. According to this method, 24 sets of samples were prepared (Fig 3). After the sample preparation was completed, the PVC pipe was buried in the ground; hence the ω of root-soil was relatively less affected by the atmosphere during the growth and development of the root system. Each PVC pipe was watered for 300ml every time and every 2~3 days. A transparent tent was built on the test site to avoid the influence of rain.

#### 2.2.1. Root growth and each layer distribution of *δ*

To obtain the overall appearance characteristics of the root system under different growth cycles, the whole vetiver with a growth time of 90d and 180d was soaked in water for 6 hours. After the expansive soil absorbed water and softened, the root system was rinsed with a root flusher. Then the absorbent paper was used to absorb the moisture on the root surface. Finally, a high-definition camera was used to take pictures. Meanwhile, the 30cm high soil is divided into six layers according to 5cm layer with a fine wire saw, and then the six layers of soil column were soaked in water for 3 hours. After the expansive soil absorbs water and softens, the solution of root-soil was passed through a 0.25 mm sieve; simultaneously the root system on the sieve was rinsed with a root flusher. The absorbent paper was used to absorb the moisture on the root surface, and finally, the roots were weighed to obtain the weight of each layer of roots. The *δ*of each layers was calculated according to formula (2). Where *υ* is the volume of each layer. Because the diameter of the PVC pipe is known, the *δ* can be obtained.

$$\delta =\frac{m}{\upsilon }$$ (2)

Where *δ* is the root content; *m* is the mass of root; *v* is the volume of soil.

#### 2.2.2. Strength test of planted soil

**Fig 4:**
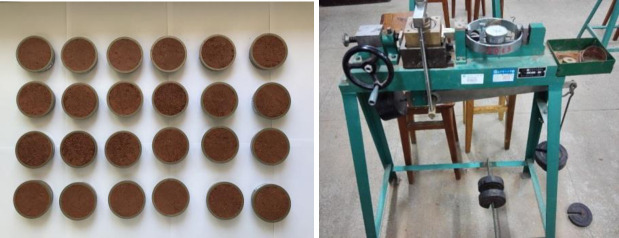
Planted soil composite with ring knife sampling and direct shear apparatus.

After 90 d and 180 d, the distribution pattern of the root was observed. The sampling position of the ring cutter was determined firstly, and upper stems and leaves were cut off with scissors. The diamond saw and scissors were used to cut the root-soil composite into soil layer at the height of 5 cm. Then the soil samples were cut with a ring-knife to obtain the root-soil composite samples. As the soil sample was 20cm, and the soil layer was 50cm, the root beyond the ring knife was carefully cut with scissors. The earth on the surface of the ring knife is then cut flat with a geotechnical knife. Furthermore, the soil cake was placed in a stripper with a confinement constraint to sample with a ring cutter. 2~3 samples were obtained from each layer, and the ω was measured. The ring cutter was a diameter of 61.8mm and a height of 20mm. Therefore, the volume (*υ*) of the soil sample can be calculated. The specimen was subjected to a direct shear test (Fig 4) according to the Trade Standard of P.R China (SL237-021 1999). In the test, four predetermined values of vertical pressure (*σ*_n_), 100,200,300, and 400kPa, respectively, were applied on four specimens for 90d and 180d. The shear rate of displacement was 0.8mm/min, and the specimen was sheared to failure or relative lateral displacement reaches 6mm within 10 to 15 min. After the test, the soil sample for the shear test was immersed in clean water for 2 to 3 hours, then the solution of root-soil was passed through a 0.25 mm sieve. The roots of the surface of the filter were rinsed with root flushers and collected to obtain the mass of roots *m*. The *δ* of the shear sample was calculated according to formula 2). At the same time, the drying method is used to test the moisture content of each layers. The distribution density was plotted for all studied independent variables (i.e. *c*, *φ*, *ρ*_d_, *δ*) to check for normality. Kruskal-Wallis tests were carried out to infer statistical differences between the non-normally distributed variables and the two treatments (i.e. fallow and planted) while ANOVA tests were implemented for the normally distributed variables at 95% and 99% confidence levels. The same tests were used to find statistical differences between each independent variable and the tested water content and density, respectively. Where statistically significant differences were encountered, the differences within the groups were evaluated by means of Wilcoxon tests and t-tests for the non-normal and normally distributed variables, respectively. Effects derived from the treatment (i.e. fallow or planted), water content (*w*), root content (*δ*) and mean density (*ρ*_d_) on the parameters (*c*: cohesion and *φ*: angle of internal friction) were evaluated by means of Pearson's correlation tests. All statistical analyses were carried using the statistical software SPSS.

#### 2.2.3. Strength test of grafted soil

The root system of vetiver with a growth period of 365d was collected and washed, and then it was cut into a length of about 70 mm. Next, roots with a diameter of 1mm were selected. Then the expansive soil was put in the oven after air-drying 12h, and crushed through the 2mm sieve. To maintain consistency with the data of the direct shear test, soil samples of five with different ω of 16%, 18%, 20%, 22%, 24%, respectively, were placed in the foam box and sealed in 24h. The amount of water to be added can be calculated by the formula (3).

$$m_{w}=\frac{m_{0}}{1+0.01w_{0}}0.01(w-w_{0})$$ (3)

Where *m*_w_ is the amount of water to be added, *m*_0_ is the mass when soil in air dry moisture, *w*_0_ is air-dry moisture content of the soil, *w* is the water content of the soil.

**Fig 5:**
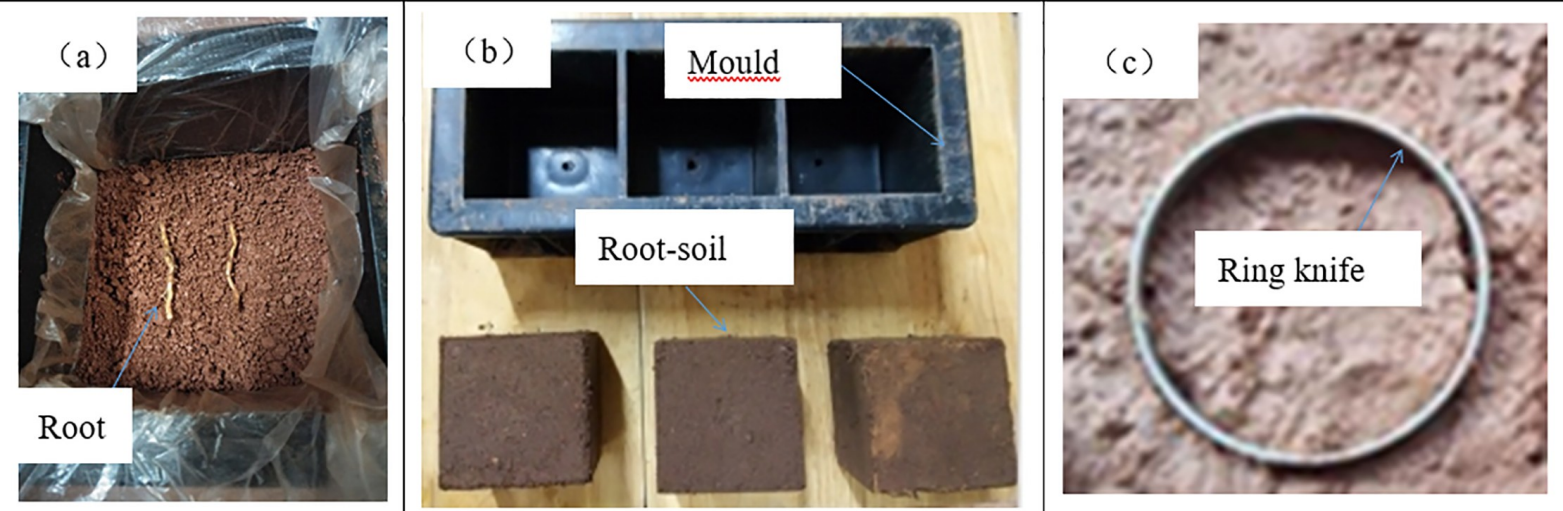
Making process of grafted soil with different root content (*δ*, g•1000cm^-3^). (a) Vetiver roots were added to the soil; (b) The root-soil sample was formed and demoulded; (c) Sampling with a ring knife.

Further, the 100mm × 100mm × 100mm cube mold was filled with soil. The degree of compaction is controlled by controlling the quality and thickness of each layer of soil. The compaction degree of 90% was adopted. The root system was placed in the mold in parallel and compacted in layers according to *δ*. The *δ* were 0.8g•1000cm^-3^, 1.6g•1000cm^-3^, 2.4g•1000cm^-3^ and 3.2 g•1000cm^-3^, respectively. Samples were removed from the mold after compaction. Finally, the ring cutter was placed on the root distribution part, i.e. vertical direction, and pressed into the cube to obtain the grafted sample slowly. As shown in Fig 5, the root system was vertically distributed in the sample. The direct shear test was carried out on the samples refer to section 3.2.2 for the specific test method.

## 3. Results and discussion

### 3.1. The root shape of vetiver and *δ* of different layer

**Fig 6:**
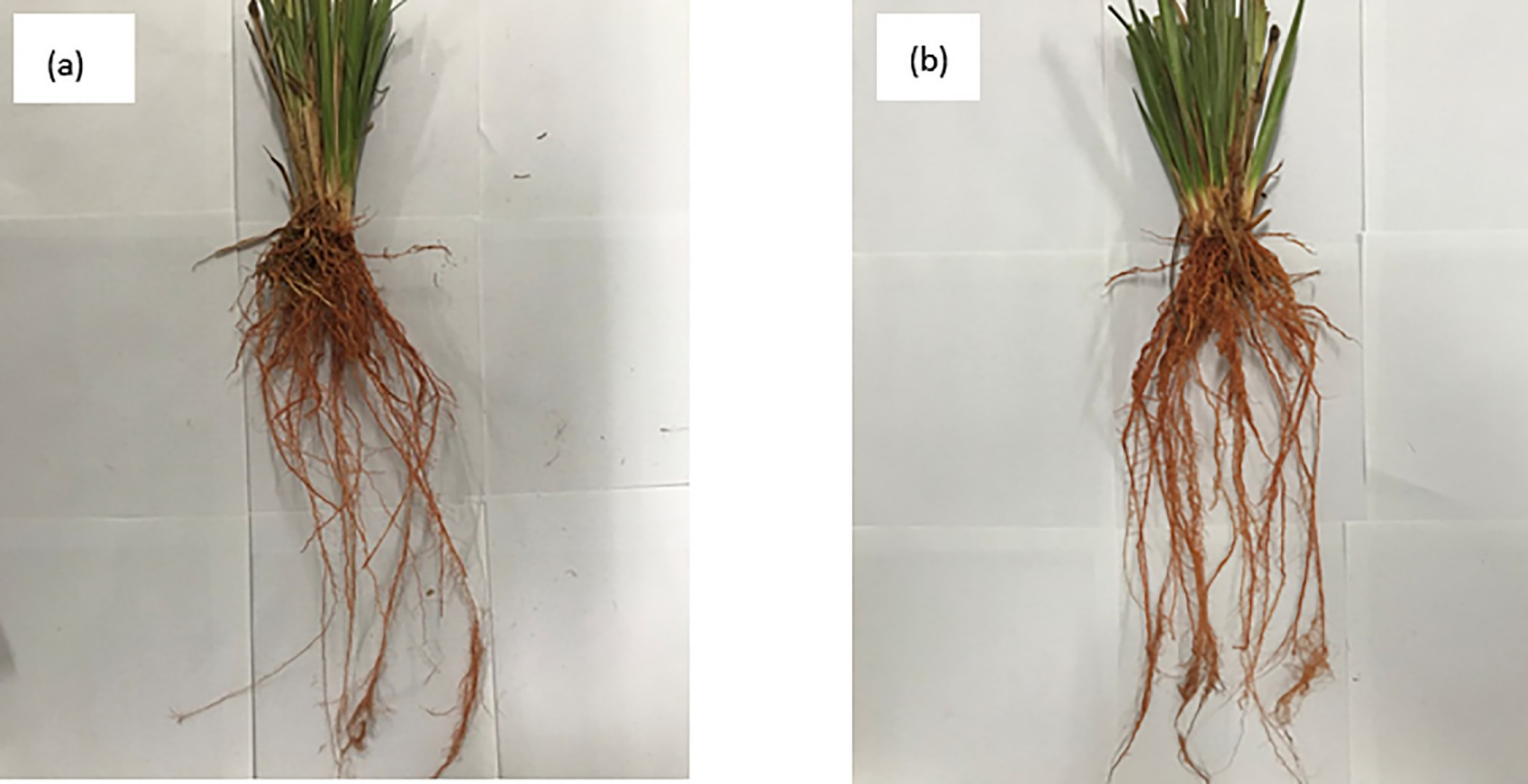
Root shape of vetiver. (a) After 90d of growth in PVC; (b) After180d of growth in PVC.

**Fig 7:**
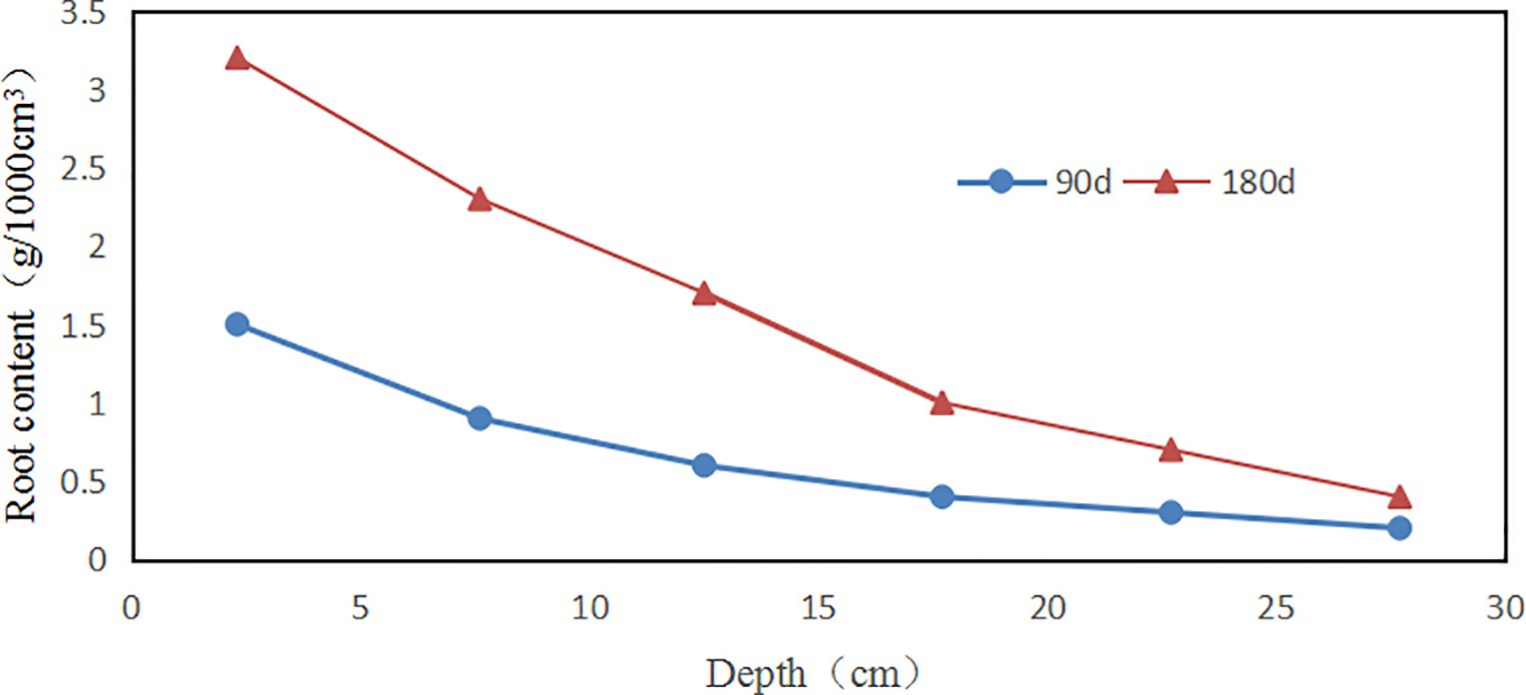
Mean root content (*δ*,g•1000cm^-3^) versus depth(cm) for vetiver with different growth time(day): The circle is 90 days; and the triangle is 180 days.

The growth of vetiver grass was observed in the vetiver culture container, and the growth and development of roots were counted. The appearance and growth distribution of roots grown for 90d and 180d are shown in Fig 6. The root diameter of the 90d vetiver in the range of 0~20cm is mostly more than 1mm, while the root diameter is mostly less than 1mm in the depth range of 20~30cm. In addition, the root depth of vetiver grass with the same depth range of 180d is thicker than that of 90d vetiver root. Fig 7 shows that the amount of root coefficient regularly decreases as the soil layer´s depth increases, which is the same as the Li et al. [26].

### 3.2. Effect of *δ* on the strength parameters of expansive soil

#### 3.2.1. Effect of *δ* on the strength parameters of planted soil

The growth time led to significant differences in terms of the root content (*δ; Z* = -2.08 *p* < 0.05, Table 2, Fig 7), where the 180d generally presented higher *δ*. Moreover, a similar pattern was seen for *δ* between different *w*(*χ*^2^ = 12.38 *df* = 5 *p* < 0.05), the *δ* was highly negatively correlated with the *w* (r = -0.48). The *δ* did not show significant differences between the considered height(*χ*^2^ = 9.34 *df* = 5 *p* >0.05), and the *δ* was highly negatively correlated with the height (r = -0.60).

**Table 2:** The direct shear test results of root-soil with different growth time in different height.

Height	Category	*ρ*_d_ (g•cm^-3^)	*w* (%)	*δ*(g•1000cm^-3^)	Growth time	*C*(kPa)	φ (°)	ΔC(kPa)	Δφ (°)
0~5 cm	Fallow soil	1.96	17.5	—	—	78.61	6.67	—	—
	Root-soil	1.92	16.8	1.52	90d	123.63	8.53	45.02	1.86
				1.37	90d	116.15	6.91	37.54	0.24
		1.99	17.6	3.39	180d	203.21	16.56	124.6	9.89
				3.67	180d	260.42	23.9	181.81	17.23
5~10cm	Fallow soil	1.75	18.1	—	—	61.6	9.54	—	—
	Root-soil	1.70	17.7	1.15	90d	84.06	9.65	22.46	0.11
				1.02	90d	85.03	9.7	23.43	0.16
		1.71	17.9	2.67	180d	153.05	13.1	91.45	3.56
				2.58	180d	124.81	12.68	63.21	3.14
10~15cm	Fallow soil	1.67	18.3	—	—	53.05	7.55	—	—
	Root-soil	1.65	18.0	0.77	90d	60.64	8.65	7.59	1.1
				0.82	90d	65.88	7.06	12.83	-0.49
		1.62	18.7	1.87	180d	97.86	16.62	44.81	9.07
				1.98	180d	111.66	15.42	58.61	7.87
15~20cm	Fallow soil	1.95	19.2	—	—	81.39	8.81	—	—
	Root-soil	1.91	18.5	0.63	90d	103.42	8.87	22.03	0.06
				0.60	90d	97.86	8.64	16.47	-0.17
		1.94	18.9	0.95	180d	198.93	16.95	117.54	8.14
				0.87	180d	188.87	19.67	107.48	10.86
20~25cm	Fallow soil	1.76	19.1	—	—	45.56	11.62	—	—
	Root-soil	1.71	18.8	0.37	90d	57.43	10.6	11.87	-1.02
				0.35	90d	57.75	10.72	12.19	-0.9
		1.74	19.3	0.67	180d	116.58	12.17	71.02	0.55
				0.68	180d	111.23	14.65	65.67	3.03
25~30cm	Fallow soil	1.73	21.2	—	—	23.85	11.48	—	—
	Root-soil	1.67	20.6	0.22	90d	35.94	12.31	12.09	0.83
				0.18	90d	38.72	11.29	14.87	-0.19
		1.71	20.9	0.53	180d	76.15	13.89	52.3	2.41
				0.47	180d	81.82	16.52	57.97	5.04

*δ*: root content(g•1000cm^-3^), *C*: cohesion(kPa), φ: internal friction angle(°), Δ*C*: the increment of cohesion(kPa), Δφ: the increment of internal friction angle (°), *ρ*_d_: mean density(g•cm^-3^), *w*: mean water content(%)

The *c* did not show significant differences between vegetated and fallow treatments (*F* = 2.66 *df* = 28 *p* = 0.11, Table 2), but show significant differences between different *δ*(*F* = 7.64 *df* = 24 *p*<0.05). In terms of *c*, it showed significant differences between the different mean density (*F* = 8.77 *df* = 14 *p*< 0.01), where the high density generally presented higher *c*. Also, the *c* differed significantly between the different *w* (*F* = 26.52 *df* = 17 *p* < 0.01). Additionally, *C* showed significant differences between height (*F* = 3.50 *df* = 5 *p*<0.05). The *c* was highly positively correlated with the *δ*(r = 0.77) and the mean density(r = 0.62). However, the *c* was highly negatively correlated with the *w* (r = -0.42) and the height(r = -0.47).

As shown in Table 2, the *c* of of 90d on 0~5cm, 5~10cm, 10~15cm, 15~20cm, 20~25cm and 25~30cm increased by 52.5%, 37.2%, 18.5%, 23.65%, 26.3%, 56.5%, respectively. The *c* of of 180d on 0~5cm, 5~10cm, 10~15cm, 15~20cm, 20~25cm and 25~30cm increased by 194.9%, 125.5%, 97.5%, 138.25%, 150%, 231.2%, respectively. It predicted that the root system could improve soil *c*, and 180d is more obvious than 90d. Combined with Fig 7, it showed that the increase of *c* is positively related to the *δ* in each depth. However, under each growth time, the *c* of the top layer and the bottom layer increases obviously, which may be the *δ* is not the only factor affecting the *c*, and there is an optimal *δ*. Firstly, in the process of root growth, the surrounding soil will be squeezed. The *δ* in the top layer is large, and the root diameter is large; thus the squeezing effect is obvious [27], which makes the soil more compact and thus increases the soil´s strength. In addition, the top layer contains a large number of roots, the root system absorbs water, and the vegetation evaporates strongly. The matric suction of soil increases, and the intensity increases [28]. Although the bottom soil has less *δ*, the initial compaction degree of soil is small, which can produce extrusion to the surrounding soil. Moreover, more capillaries can fill the gap between soil particles, and the strength increases more obviously.

The *φ* also did not show significant differences between vegetated and fallow treatments (*F* = 0.98 *df* = 28 *p* = 0.33, Table 2). Additionally, *φ* did not show significant differences between height (*F* = 0.27 *df* = 5 *p* = 0.92). But show significant differences between different *δ*(*F* = 4.75 *df* = 24 *p*<0.05). The mean density led to significant differences in terms of the φ (*F* = 5.98 *df* = 14 *p* <0.01, Table 2). Moreover, the *φ* (Table 2) also presented statistically significant differences with the *w* (*F* = 7.64 *df* = 17 *p* <0.01). The φ was positively correlated with the *δ*(r = 0.55) and the mean density(r = 0.15). Additionally, φ was low positively correlated with the *w* (r = 0.23).

The φ of 90d on 0~5cm, 5~10cm, 10~15cm, 15~20cm, 20~25cm and 25~30cm increased by 15.7%, 1.5%, 4.1%, - 0.6%, - 8.3% and 2.8% respectively. The φ of 180d on 0~5cm, 5~10cm, 10~15cm, 15~20cm, 20~25cm and 25~30cm increased by 203.3%, 30.1%, 112.2%, 107.9%, 15.4% and 32.5% respectively. In each depth, the increase of *φ* of 90 d is not obvious, which is the same as the test result of Yu et al. [29], but the φ of the 180d root-soil composite is larger than that of fallow soil.

A possible explanation for this might be that the 90d root-soil composite has low *δ*, and the root tensile strength is less than the maximum static friction force. As the shear displacement increases, the root is pulled off, and almost no sliding friction is generated. The 180d root-soil composite has high *δ*, and the tensile strength of the root is greater than the maximum static friction. With the increment of shear displacement, the root is pulled out to produce sliding friction, which improves *φ* of the soil. Therefore, both the *c* and *φ* of the 180d root-soil composite are significantly higher than those of fallow soil.

**Fig 8:**
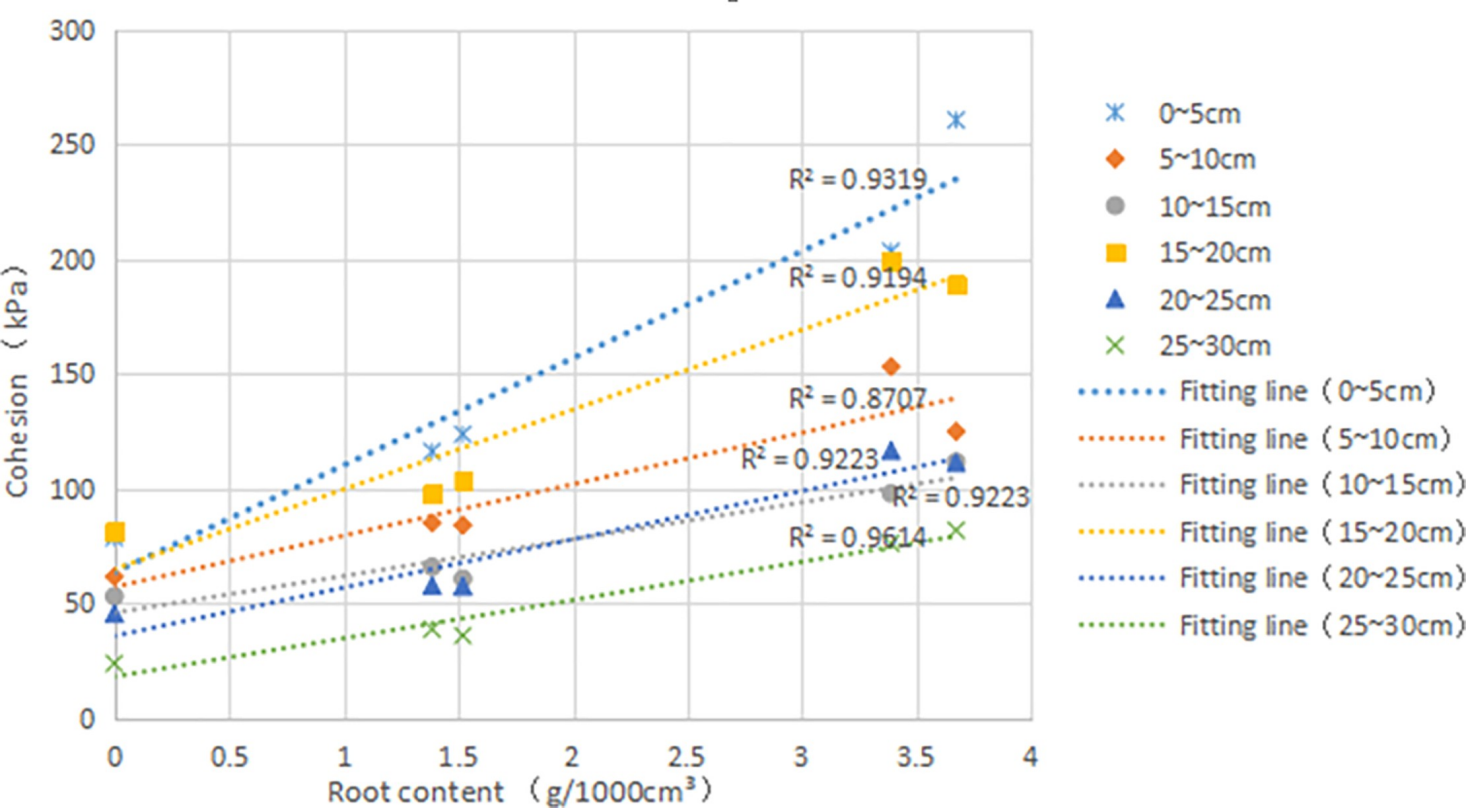
Cohesion (*c*, kPa) versus root content (*δ*, g•1000cm^-3^) in different depth(cm): R^2^, goodness of fit.

Fig 8 is the relationship curve between *c* and *δ*. It is found that the *c* of root-soil increases with the increase of *δ* at each depth. This result indicated that the *δ* affects on the *c* of the sample. It can also be found from the figure that the fitting slope of each layer is not the same. If the slope of the line is large, indicating that the *δ* has more influence on the *c* of this layer than other factors. Firstly, a high planting density could prevent the roots from sliding from the soil, increasing resistance to shear [30]. 0~5cm and 15~20cm have high density; hence the two layers have better reinforcement than other layers. Secondly, for a certain soil engineering, the structure and density of the soil will not change too much in a local area. And the influence of the change of ω on the strength may be more significant than other factors [31]. Simultaneously, the standards for making the density of the two layers of 0~5cm and 15~20cm are the same. Thus the small changes in the density can be ignored, but the slope of the fitting straight line is different, and the *ω* of the two layers is different. The possible reason is that the increase in *ω* leads to a decrease in strength. Since the 0~5 layer is close to the surface, this layer is greatly affected by the atmospheric environment, and the *ω* varies greatly. The soil layer in deep is less affected by the atmosphere, and the change of *ω* is small, thus the linear slopes of 5~10 and 20~25, 10~15 and 25~20 are not significantly different. The next indoor test will deeply analyze the influence of *ω* on *c*.

**Fig 9:**
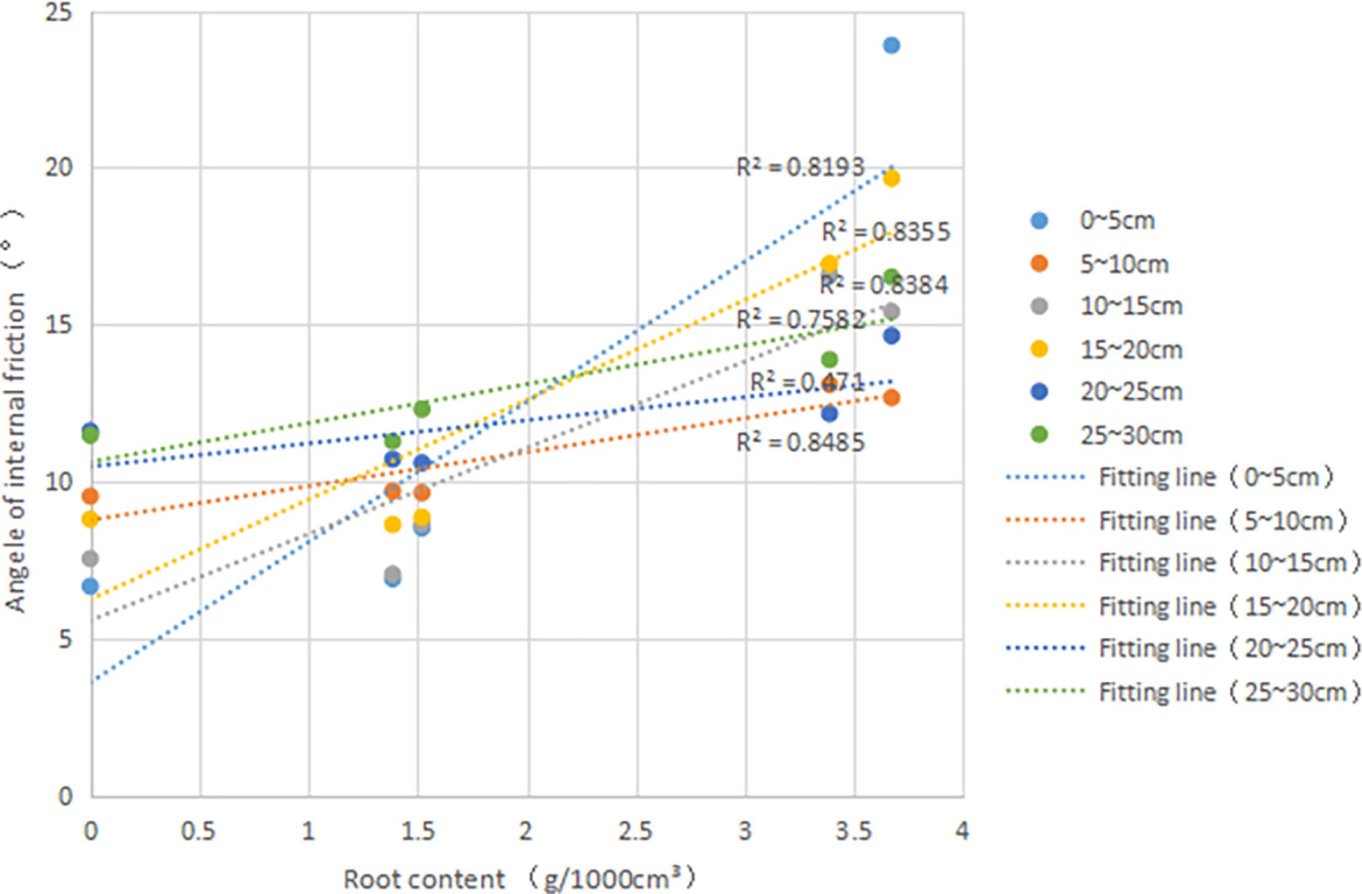
Internal friction angle (*φ*,°) versus root content (*δ*, g•1000cm^-3^) in different depth(cm): R^2^, goodness of fit.

It is evident that the *φ* of root-soil increases with the increase of *δ* (Fig 9), indicating that the *δ* affects on the *φ* of the sample. If the slope of the straight line is large, it means that the *δ* has a significant impact on *φ*. It can be seen that the slopes of the 0~5 and 15~20 layers are larger than the slopes of the other layers, and the slopes of the two layers are not the same; it is also possible that the *ω* is different. An increase in the *ω* will weaken the root´s enhancement of the *φ*.

The growth time led to significant differences in terms of the Δ*c* (Z = -4 p < 0.01, Table 2). Moreover, a similar pattern was seen for Δ*c* between water content (*χ*^2^ = 11.26 df = 5 p < 0.05), *δ* (*χ*^2^ = 11.94 df = 3 p < 0.01), fallow and vegetated(*χ*^2^ = 7.19 df = 1 p < 0.01), and different height(*χ*^2^ = 12.40 df = 5 p < 0.05). The Δ*c* was highly positively correlated with the *δ*(r = 0.78) and the growth time(r = 0.76). However, the Δ*c* was negatively correlated with the *w* (r = -0.16) and the height(r = -0.26).

**Fig 10:**
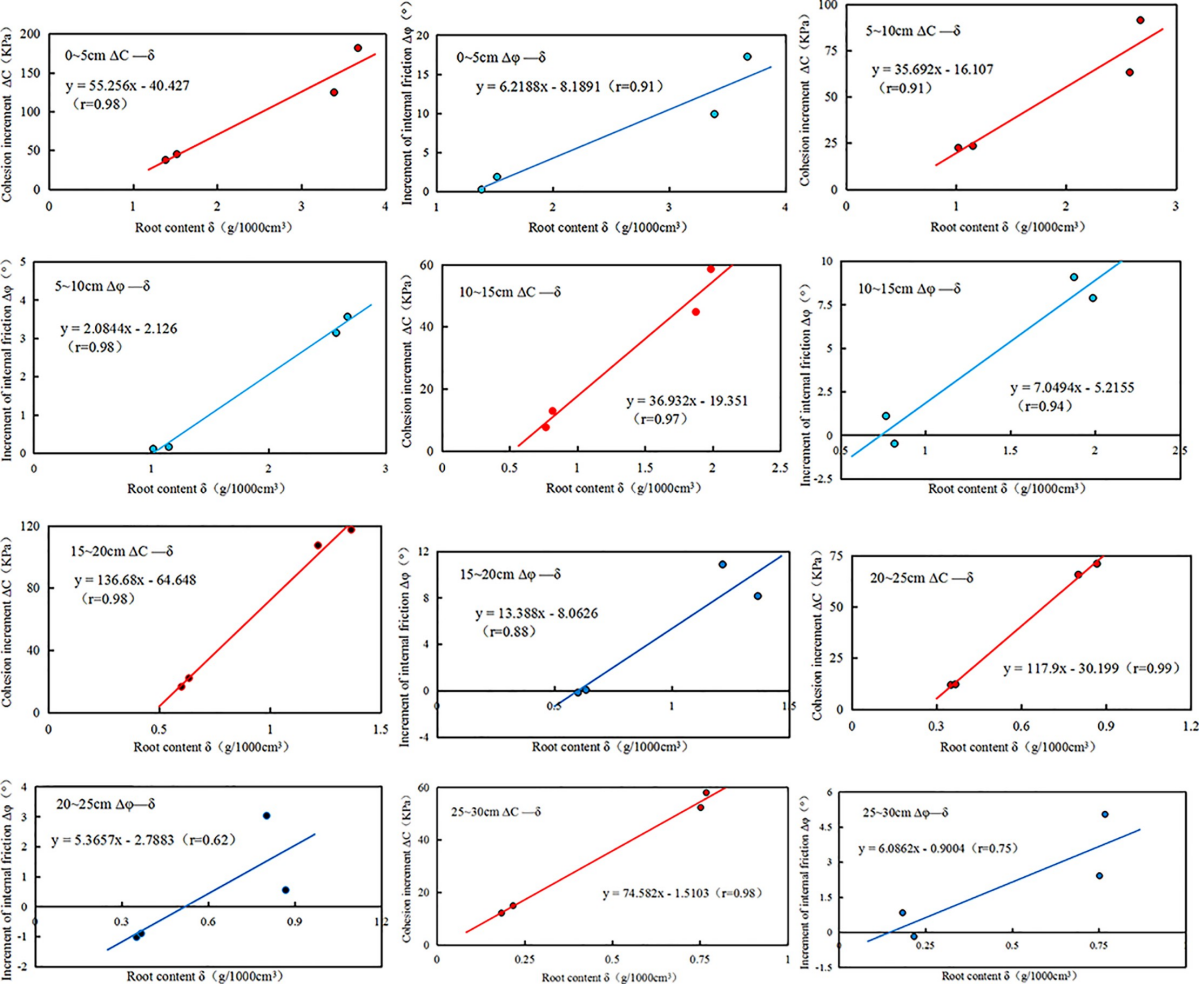
The increment of cohesion(Δ*c*, kPa) and the increment of internal friction angle (Δ*φ*, °) versus root content (*δ*, g•1000cm^-3^) in the different depth(cm): r, the correlation coefficient; y, the fitted equation of the line.

Fig 10 shows that the Δ*c* increases with the increment of *δ*. The correlation coefficient was 0.91 at the minimum and 0.99 at the maximum. The higher slope of Δ*c* and *δ* fitting curve, the more significant the influence of *δ* on soil. Therefore, the results showed that the root system of the vetiver could improve *c*.

In each depth, the Δ*c* increases with the increment of the *δ*, which is different from other scholar´s research. Theoretically, the Δ*c* can´t increase infinitely with the *δ* in each depth. When the *δ* exceeds a particular value, Δ*c* will decrease with the increase of *δ*. The main reason for this test result is that the growth and development time of the experimental planting vetiver is short; the overall *δ* is small and does not reach the optimal *δ*.

The growth time led to significant differences in terms of the Δ*φ* (Z = -4.10 p < 0.01, Table 2). Moreover, a similar pattern was seen for Δ*φ* between *δ* (*χ*^2^ = 9.97 df = 3 p < 0.05), fallow and vegetated(*χ*^2^ = 7.19 df = 1 p < 0.01). The Δ*φ* did not show significant differences between the considered height (*χ*^2^ = 2.88 df = 5 p >0.05), and water content (*χ*^2^ = 9.97 df = 3 p < 0.05). The Δ*φ* was highly positively correlated with the *δ*(r = 0.72) and the growth time(r = 0.72). However, the Δ*φ* was negatively correlated with the *w* (r = -0.11) and the height(r = -0.26).

In addition, the correlation coefficient between the Δ*φ* and the *δ* is also approximately linear. The highest correlation coefficient is 0.98, the lowest is 0.62, but the correlation coefficient of the soil layer in the range of 0~15cm of high *δ* is significantly higher than that in the field of 15~30cm of low *δ*. It shows that the linear relationship of Δφ and *δ* in soil with high *δ* is reliable, while that of Δφ and *δ* in soil with low *δ* is not obvious.

A possible explanation for this might be that the *δ* of 0~15cm is high, and the root diameter is thick; the effective contact area between the root system and soil is larger than that of the 15~30cm soil layer. Thereby the *φ* is much improved. While the *φ* of low *δ* soil layer is almost no enhanced, hence the correlation coefficient of fitting curve 0~15cm is larger than that of 15~30cm. Another possible explanation for this is that the root system grows free in a natural growth state. While the limitation of the size of the direct shear test specimen, the root-soil in the ring cutter has the possibility of the same *δ* but different root distribution, number, and diameter. Therefore the test results have certain discreteness.

#### 3.2.2. Effect of *δ* on the strength parameters of grafted soil

**Fig 11:**
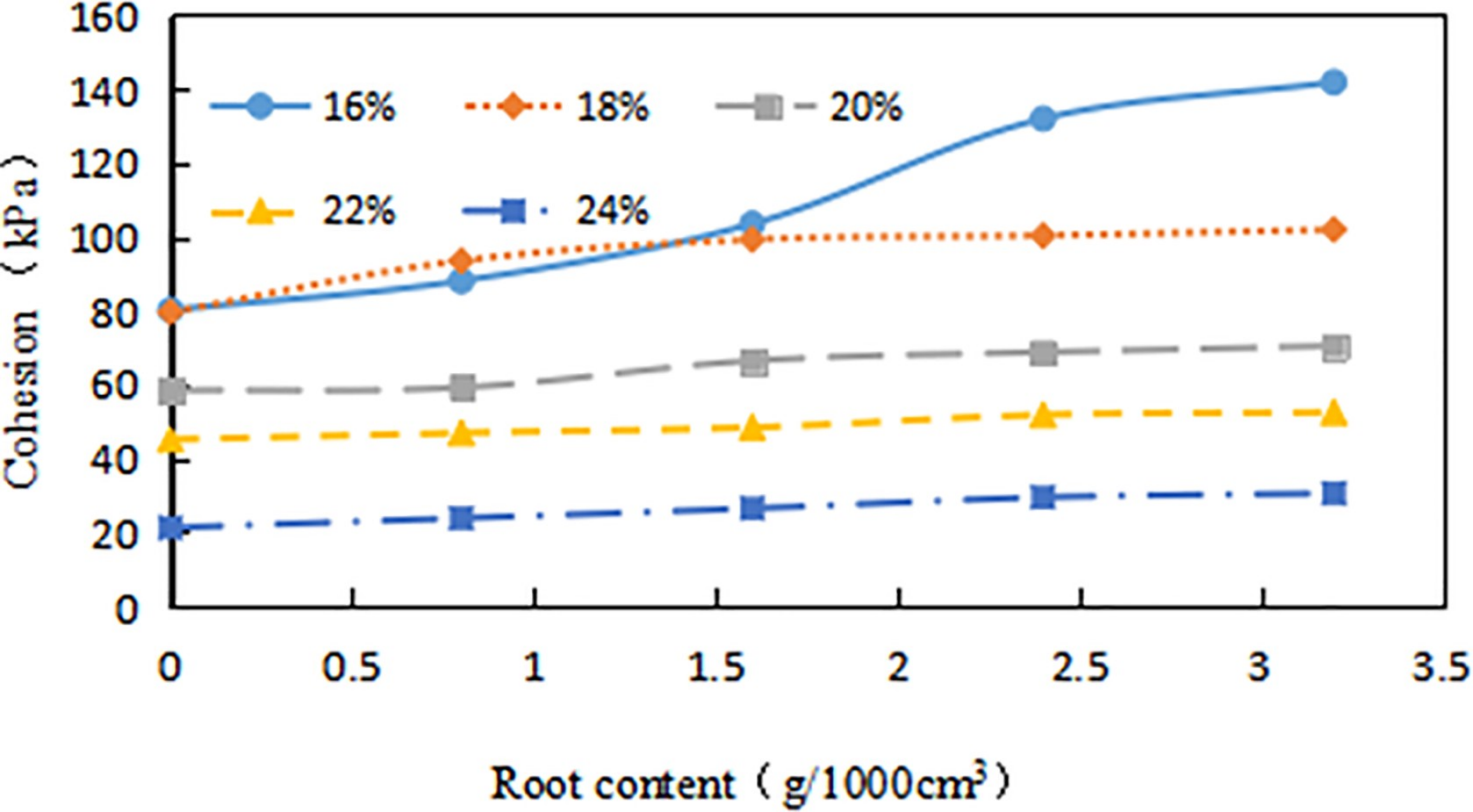
Relationship between cohesion (*C*, kPa) and root content (*δ*, g•1000cm^-3^): The percentage in the figure is the water content (*ω*, %).

The *C* of different *ω* gradually increased with the increase of *δ* (Fig 11). When the *ω* was high, the *δ* had little effect on the *C*, and the growth was slow. The lower the *ω*, the greater the increase in *C* as the *δ* increases. When the ω are 16%, 18%, 20%, 22% and 24%, the average *C* of the root-soil is increased by 53.39%,38.7%,42.3%, 34.49%, and 33.6%, respectively compared with the fallow soil. Especially, when the *δ* is 3.2g•1000cm^-3^, the *C* is increased by 85.22% compared with fallow soil. It is fully proved that the existence of vegetation roots plays a key role in increasing *C*. But when the *ω* is large, the added effect of *C* with the increase of *δ* is not obvious.

**Fig 12:**
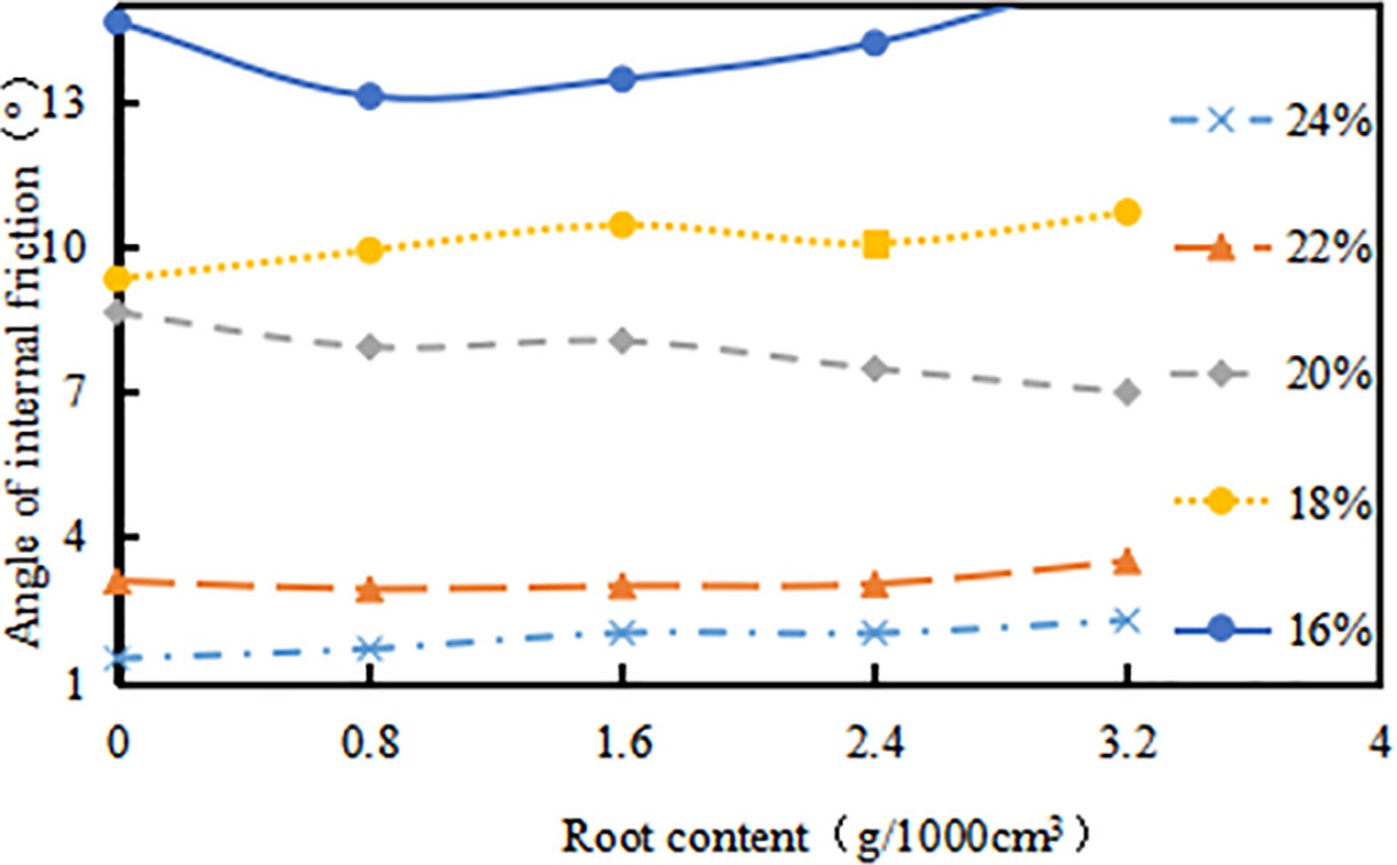
Relationship between angle of internal friction (*φ*, ^o^) and root content (*δ*, g•1000cm^-3^): The percentage in the figure is the water content (*ω*, %).

When the *δ* increases, the curve of *φ* versus *δ* shows a slight upward trend (Fig 12). But the variation range is not obvious, for example, the *δ* increased from 0.8g/1000cm^3^ to 3.2g/1000cm^3^, and the *φ* of the root-soil increased by 6.2%, 3.9%, 1.6%, 22.2%, 0% respectively when *ω* were 16%, 18%, 20%, 22%, 24%. It is indicated that the *δ* has little effect on the *φ* of the expansive soil. In addition, when the *ω* of the sample exceeds 20%, the *φ* slowly increases with the increment of root content, which shows that the root has no significant effect on the *φ* when the *ω* is lower than 20%.

Cheng et al. [32] studied the strength rule of composite soil under synthesize, inclined and vertical roots, and the strength of composite soil under vertical conditions is more greater than that under inclined conditions. In this paper, most of the roots are inclined. To make the sampling easier, the root system is vertically distributed. Liu et al. [33] found that the composite soil´s strength increased with the increase of the diameter. In this study, most of the root diameter was about 1mm, and the strength of grafted soil in 365d should be greater than 180d, but the research results showed that the strength rule of grafted soil in 365d was similar to 90d, indicating that the effect of grafted root to enhance the strength of the expansive soil was not significant. The root system enhances the strength of expansive soil. Firstly, due to the biological activity of the root system, such as the transpiration of vegetation, the matric suction of soil increases. The research shows that the matric suction of soil can be increased by about 20%. But grafted root does not effect. In addition, in the process of root growth, the root system produces extrusion pressure on the surrounding, which makes the soil denser and increases the strength of the soil. Finally, the wrapping effect of root system, cross winding, improves the strength of the soil. Therefore, the root system´s effect on soil strength was better in 180 days than in 365 days.

**Fig 13:**
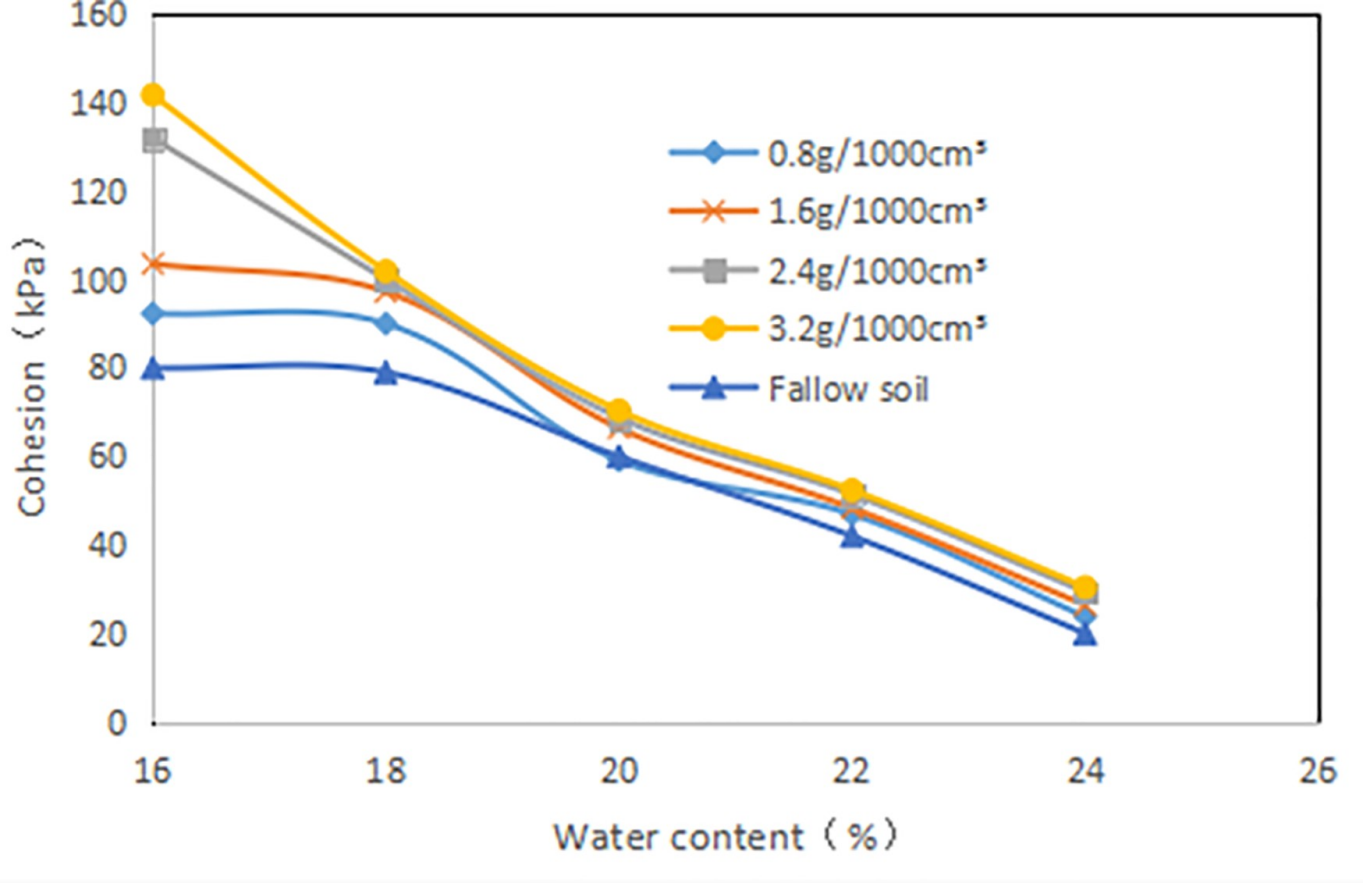
Relationship between cohesion(*C*, kPa) and water content (*ω*, %).

It is clear that as the *ω* increases, the *C* gradually decreases (Fig 13). Compared with fallow soil, the reduction in *C* of the sample increase with the increase of *δ*. For example, the *ω* increased from 16% to 24%, and the *δ* of 3.2g/1000cm^3^ and 0.8g/1000cm^3^ decreased by 78.5% and 74.2% respectively.

**Fig 14:**
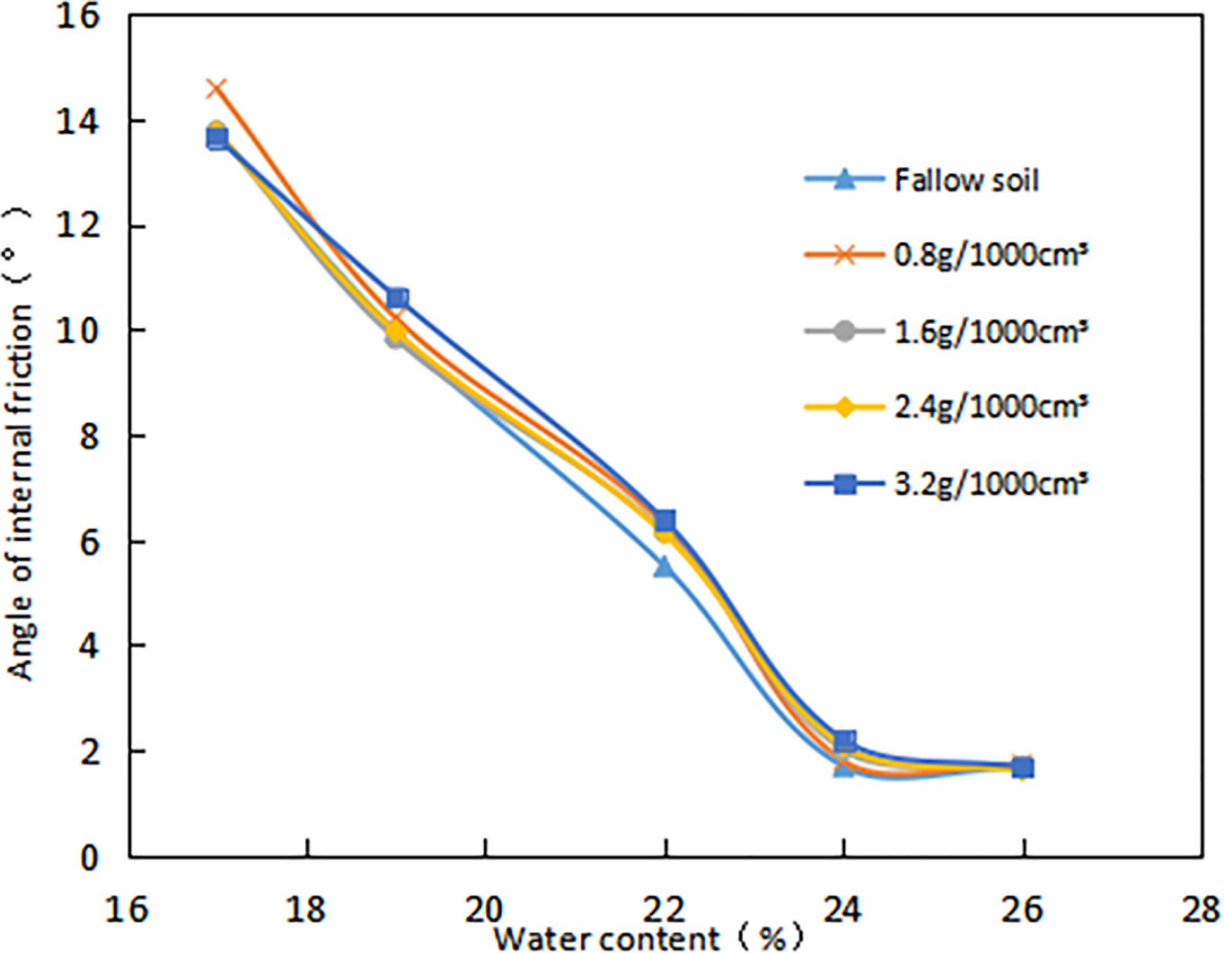
Relationship between angle of internal friction(φ, °) and water content (ω, %).

It is indicated that as the *ω* increases, the *φ* gradually decreases (Fig 14). The *ω* increased from 16% to 24%, and the *δ* was 0.8g/1000cm^3^, 1.6g/1000cm^3^, 2.4g/1000cm^3^, 3.2g/1000cm^3^, which decreased by 88.3%, 87.6%, 88.3% and 87.5% respectively. In summary, the *C* and *φ* of root-soil are greatly affected by *ω*.

In expansive soil areas, most of the expansive soil is used as the foundation or basic material for engineering. Due to climate changes (such as rain, etc.), the *ω* of the expansive soil will inevitably lead to changes in its strength [31]. In addition, the article believes that the strength of expansive soil decreases with the increase of *ω*, and the decrease of *C* is more obvious than the decrease of *φ*. For root-soil, we found a similar rule, indicating that the strength of root-soil decreases with the increase of *ω*, but the decrease in φ is more evident than the decline in *C*, and the difference between the two decreases is not massive. Firstly, the root-soil bonds may change with the *ω* [34]. As a result, the mechanisms of root failure [35] can vary [36] along with the amount of energy conferred to the soil by the root system [35]. The maximum energy is thought to be provided when the roots break [34, 37]. Yet, when the soil is extremely saturated, roots will be more likely to pull-out [34] as a consequence of the soil's physical consistency loss [38] and derived soil-root bond loss [34].

#### 3.2.3. Comparative analysis of strength parameters of different soil

**Fig 15:**
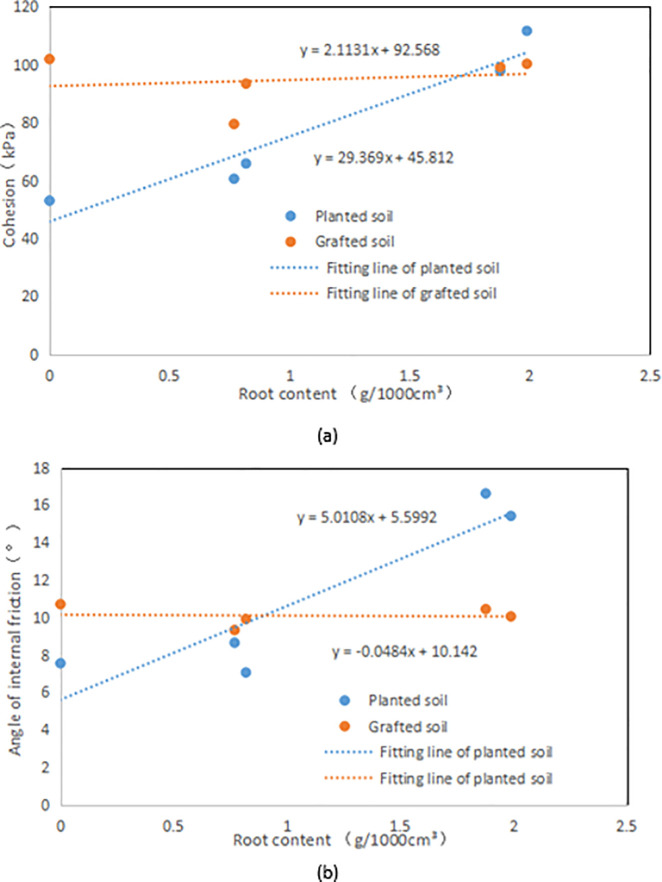
The influence of root content (*δ*, g.1000cm^-3^) on the shear strength parameters of different soils: (a) Effect of root content (*δ*, g.1000cm^-3^) on cohesion (*C*, kPa); (b) Effect of root content (*δ*, g.1000cm^-3^) on angle of internal (*φ*, ^o^).

Fig 15 is showing the relationship between strength parameters and *δ* for different soil. Since the compaction of the 10~15cm layer is the same as that of grafted soil, and the *ω* of the two is similar, they are compared and analyzed. The slope of the fitted straight line between the shear strength parameter and *δ* of the planted soil is greater than that of the grafted soil. It shows that the strength parameter of the root-soil with the addition of grassroots does not increase significantly with the increase of *δ*, while the *δ* of the planted soil has a significant effect on the strength parameters of the root-soil.

It is clear that the root reinforcement of grafted soil is weaker than the root reinforcement of the planted soil. The reason is that the growing grass root system can bind the individual grains of the soil together. At the same time, the huge and developed fibrous root system of the plant can create a crisscross root network in the soil, which tightly binds and wraps the soil particles to form "reinforced soil". This result greatly improves the shear strength of the soil. The addition of grassroots to grafted soil is only to combine and compact the root system with the soil, and the degree of interaction between the root and soil is poor. On the other hand, grafted soil is mainly composed of vertical roots. But the roots for planted soil include vertical and horizontal roots that will improve the strength of the soil [14]. Therefore, the bonding force between the root and soil of planted soil is relatively large, and the root system can strengthen the strength of the soil obvious. This study also shows that the reinforcement effect of simulating planting roots by adding grassroots is often quite different from the actual ones.

## 4. Conclusions

In light of our observations and findings, it can be concluded that:

1. The *δ* of vetiver decreased with the increase of depth, and the *δ* increased with the growth period. The *δ* of 180d was 70.5% higher than that of 90d.
2. For planted soil, the *c* of root-soil can be increased by more than 97%, and φ can be increased by more than 15.4% after 180 days. The *c* of 90 d vetiver root system can be increased by more than 18%, and the φ can be increased by more than 1.5%. At the same depth, the strength of composite soil increases with the increase of the *δ*.
3. The *δ* and *ρ*_d_ has an impact on the shear strength parameters, and the *c* and φ both increase with the increase of the *δ ρ*_d_. Simultaneously, an increase in the *ω* will weaken the root's enhancement of the shear strength parameters. The *C* gradually decreases with the increase of depth.
4. For each layer, the Δ*c* increases with the increment of *δ*. The correlation coefficient was 0.91 at the minimum and 0.99 at the maximum. In addition, the correlation coefficient between the increment of φ and the *δ* is also approximately linear. The highest correlation coefficient is 0.98, and the lowest is 0.62.
5. For grafted soil, the lower the *ω*, the greater the increase in cohesive force as the *δ* increases. When the ω is 16%, 18%, 20%, 22% and 24%, the average *c* of the root-soil is increased by 53.39%,38.7%,42.3%, 34.49%, and 33.6%, respectively compared with the fallow soil. When the *ω* of the sample is greater than 20% (Optimum water content), the *φ* slowly increases with increment of content of root, which shows that the root has no significant effect on the *φ* when the *ω* is lower than 20% (Optimum water content).
6. Under the condition of planted root system and grafted root system, the influence of *δ* on soil strength is different. The grafted soil can't accurately reflect the root-soil interaction of the existing root system, and actual reinforcement results were better than grafted soil.
7. For grafted soil, the strength parameters of root-soil decreases with the increase of *ω*, but the decrease in φ is more evident than the decline in *c*.

The reinforcement effect of the actual root system is not only related to root diameter and *δ*, but also related to root distribution direction, root quality, and root length. These factors is not considered in this paper and needs further research in the future.

## Supporting information

S1 Data (RAR)
